# Multi-level QTAIM-enriched graph neural networks for resolving properties of transition metal complexes

**DOI:** 10.1039/d5dd00220f

**Published:** 2025-10-15

**Authors:** Winston Gee, Abigail Doyle, Santiago Vargas, Anastassia N. Alexandrova

**Affiliations:** a Department of Chemistry and Biochemistry, University of California Los Angeles California 90095 USA santiagovargas921@gmail.com ana@chem.ucla.edu; b Department of Materials Science and Engineering, University of California Los Angeles California 90095 USA; c California NanoSystems Institute Los Angeles California 90095 USA

## Abstract

Here we evaluate the robustness and utility of quantum mechanical descriptors for machine learning with transition metal complexes. We utilize *ab initio* information from the quantum theory of atoms-in-molecules (QTAIM) for 60 k transition metal complexes at multiple levels of theory (LOT), presented here in the tmQM+ dataset, to inform flexible graph neural network (GNN) models. We evaluate these models with several experiments, including training on limited charge and elemental compositions and testing on unseen charges and elements, as well as training on smaller portions of the dataset. Results show that additional quantum chemical information improves performance on unseen regimes and smaller training sets. Furthermore, we leverage the tmQM+ dataset to analyze how QTAIM descriptors vary across different LOT and probe machine learning performance with less computationally expensive LOT. We determine that *ab initio* descriptors provide benefits across LOT, thereby motivating the use of lower-level DFT descriptors, particularly for predicting expensive or experimental molecular properties.

## Abstract 2

Here we evaluate the robustness and utility of quantum mechanical descriptors for machine learning with transition metal complexes.

## Graphical abstract

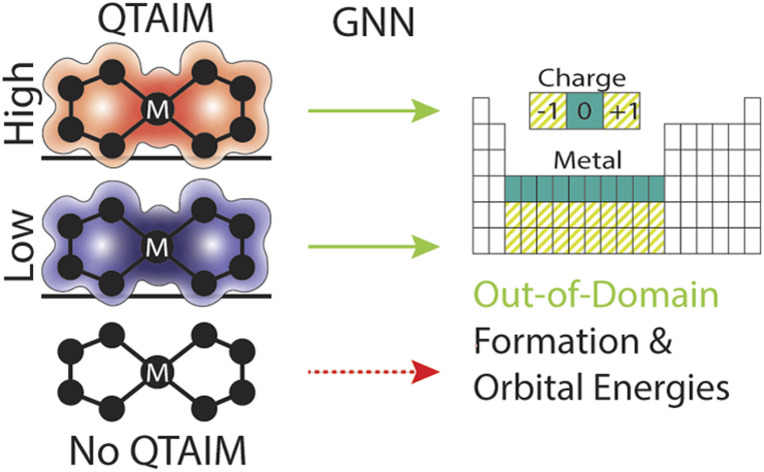

## Introduction

Modern computational chemistry relies upon Density Functional Theory (DFT).^1–5^ This methodology has facilitated the discovery and understanding of chemical processes in the domains of batteries,^6,7^ medicine,^8,9^ catalysis,^10,11^ enzymology,^12–14^ and green energy.^15,16^ At the same time, DFT is fundamentally limited to certain regimes. Systems larger than hundreds of atoms, including those with heavy elements or long-range interactions, reach the limits of DFT due to computational affordability and accuracy. Much chemistry exists beyond this range, including the extensive screening of molecules with heavy atoms or the systematic evaluation of millions of molecules in high-throughput studies. With DFT, these approaches require costly individual evaluation for each system with no notion of generalizability or transferability to new systems. Furthermore, these studies easily amount to hundreds of millions of core-hours even for libraries of small molecules.^2,17^

Machine learning (ML) offers an alternative paradigm for predicting molecular properties with rapid inference times and interpolation for systems in domain to the training set. Though chemical ML algorithms are often trained on DFT data,^18–21^ unlike DFT, they allow scientists to characterize new molecules rapidly.^22–24^ In addition, modern machine learning approaches offer interpretability methods that can uncover novel physical properties by analyzing trends over entire datasets.^13,25–28^

The power of ML models depends on a relevant training set. Training sets are often laboriously compiled from the literature or *via* heuristics when applying ML to a new chemical domain. Accordingly, datasets often include only certain labels, descriptors, or chemical motifs. For example, the most popular chemical benchmark dataset, QM9 contains up to only 9 heavy atoms limited to carbon, nitrogen, oxygen, or fluorine.^4^ ZINC15, a dataset of commercially available compounds for drug discovery, is larger and more diverse but with a narrow set of cheaper properties, such as water–octanol partitioning (log *P*), molecular weight, and size.^29^ Therefore, chemists interested in applying ML continue to develop new datasets of molecules with varied charges, spins, and sizes for broad applications and spanning the periodic table. There are a few notable examples, including OMat^2^ and the Materials Project^30^ in periodic systems, and OMol25 (ref. 1) and MPcules^5^ for molecular systems. Here we choose to build upon the tmQM^31^ dataset, and its refined variants tmQMg^32^ and tmQM-wB97MV.^33^ These datasets provide an excellent repository of transition metal complexes with a significant set of computed properties, including formation energies and orbital energies. The complexes each have a single-metal center from across the entire d block with a variety of ligands.^31^ The dataset also includes complexes with varying charge, an important variable not often swapped in chemical datasets.

In order to perform machine learning with these transition metal complexes, we consider how to represent their chemical information. Common representations of organic molecules include SMILES strings,^34^ vectors of DFT descriptors, graphs, and more recently, hypergraphs,^35^ but transition metal complexes are more complicated to represent due to the d orbitals of the metal exhibit bonding behavior beyond simple two center-two electron bonds. In order to address this challenge, Kneiding *et al.* developed a natural quantum graph (NatQG) based upon natural bond orbital (NBO) analysis.^32^ This approach yields graphs featurized with *ab initio* information, but a heuristic was necessary to ensure the molecular graphs were fully connected as a single entity for each molecule. Additionally, others have recently adapted SMILES strings to operate on transition metal complexes by including structural, connectivity, and charge information and used them to train traditional cheminformatic models.^36^ Herein we propose representing transition metal complexes as graphs built upon the quantum theory of atoms-in-molecules (QTAIM), this follow previous studies where we constructed molecular graphs using this representation.^13,37^ The quantum theory of atoms-in-molecules (QTAIM)^38^ rigorously ascribes the electron density of a molecule into its respective atoms by partitioning along surfaces of zero flux in the electronic density. Topological analysis of the density identifies critical points (CP) where the density is maximized in all directions, deemed nuclear CP as they occur near nuclei, whereas CP marking density maxima in two dimensions and minima in the third are bond CP. Additionally, ring CP are found where the density is minimized in two dimensions yet maximized in the third, and cage CP are local minima in the electron density. Furthermore, paths of steepest ascent are mathematically guaranteed from a bond CP to its neighboring nuclear CP, thereby outlining a unique path between nuclear CP called the bond interaction path. As such, QTAIM provides a set of paths connecting critical points which we utilize to build fully connected graphs for the transition metal complexes. Additionally, QTAIM provides various descriptors (see ‘QTAIM Features') of the density measured at the critical points which we can include in our QTAIM graph representation of transition metal complexes.

We hypothesize that including quantum chemical features in the form of QTAIM descriptors can extend model efficacy for out-of-domain predictions, perhaps even to unseen elements, charges, and molecular sizes. Many machine learning models, in particular graph neural networks, have a poor ability to generalize outside of training data,^20,21,39^ but quantum mechanical descriptors can enhance their generalizability in some cases.^40^ This work assesses performance improvements with QTAIM descriptors for general GNN architectures. These assessments include testing on small training sets and out-of-domain experiments, two areas where we believe that adding quantum information can offer model improvements. For the out-of-domain experiments, we performed several train-test evaluation studies, including training on limited charge and elemental compositions while testing on unseen elements and charges. We anticipate that additional descriptors from QTAIM will enhance the predictivity of models in these cases.

Naturally, this raises the concern of predicting computational values with other computational values from similar formalisms. We approach this concern by benchmarking ML model performance across two levels of theory (LOT) for geometries and two LOTs for electronic densities used to calculate QTAIM descriptors. This design isolates the effects of geometry LOT and density LOT on the effectiveness of quantum-informed ML models. We seek to evaluate whether QTAIM descriptors from less computationally expensive geometries and densities sufficiently provide similar benefits to the models. Furthermore, considering the dependence of QTAIM analysis on the level of theory utilized to obtain the density, we assess the variation of the QTAIM descriptors across the different levels of theory. These systematic benchmarks of QTAIM descriptors provide valuable insight to the traditional theoretical community as the first (to our knowledge) high-throughput benchmark of QTAIM across different levels of theory.

## Methods

### General molecular property graph neural networks

We previously developed a general-purpose graph neural network (GNNs) package, qtaim-embed.^19^ This code implements a host of message-passing architectures and components including attention pooling,^41^ set2set pooling,^42^ graph convolutions,^43^ mean global pooling, *etc.* The transition metal complexes herein are encoded as heterographs with separate types of nodes to represent atom-level, bond-level, and global information, thereby enabling unique edges to capture specific interactions between the node types. Molecular level information—such as spin, charge, and molecular weight—is included in the global feature vector, while information from a specific atom or bond forms a feature vector for an atom-level node or bond-level node, respectively. The specific atom and bond features are detailed in the following section, and further details about heterograph construction can be found in Section 3.1 ‘Molecular representation’ in our previous work. Following featurization, features are embedded to a fixed size vector for each node type and graphs are updated through a message-passing layer prior to global pooling. Finally, this fixed-sized representation passes through a standard, fully-connected network for property prediction.

Building on these prior developments, we implemented a host of new features to allow for improved training and evaluation. We integrated linked-memory databases (LMDBs) where data is preprocessed (converted to deep learning graph (DGL)^44^ structures, standardized, and featurized) and saved to disk. This allows users to offload the memory and compute-intensive task of preprocessing datasets to supercomputer resources. This vital development allowed for rapid training and effective GPU usage given the large number and size of molecules in the tmQM dataset.

### QTAIM features

To complement this GNN package, we implemented a high-throughput tool to compute and process QTAIM features into a form ready for machine learning. The qtaim-generator package performs density functional theory (DFT) calculations with ORCA^45,46^ followed by QTAIM analysis with Multiwfn^47^ and parses the output into a simple format for machine learning. We accordingly generate a rich set of over twenty QTAIM descriptors measured at nuclear critical points and bond critical points. The full set of features is available in Table S1 and in the original manuscript.^19^

### tmQM+ datasets

We aim to benchmark how well geometric ML models perform with quantum mechanical descriptors at different LOT. We thus constructed three datasets at various LOT derived from the original tmQM dataset and subsequent renditions. The tmQM dataset^31^ provided 86 k transition metal complexes optimized with xTB and labeled with TPSSh-D3BJ/def2-SVP single point energies. Kneiding *et al.*^32^ subsequently performed geometry optimizations upon these complexes at the PBE-D3BJ/def2-SVP level to afford a set of 60 k structures with PBE0-D3BJ/def2-TZVP single points in their tmQMg dataset. Garrison *et al.*^33^ identified and removed unphysical structures from the original dataset in their tmQM-wB97MV dataset. After similarly removing these unphysical structures, we gathered geometries available at both the xTB and PBE-D3BJ levels of theory, matched them by molecule ID and labeled both sets with HOMO, LUMO, HOMO–LUMO gap, and formation energies at the PBE0-D3BJ/def2-TZVP level of theory from the tmQMg dataset. With these geometries in hand, we performed DFT single point calculations in ORCA to obtain densities for QTAIM analysis: at a higher level of theory (PBE0-D3BJ/def2-TZVP level with DKH basis contraction) for the PBE-D3BJ optimized geometries and at a lower level of theory (TPSS-D3BJ/def2-SVP with DKH basis contraction) for the xTB optimized geometries. For comparison, a third dataset was computed with the PBE-D3BJ optimized geometries at the cheaper TPSS-D3BJ/def2-SVP level. These three tiers enable the first high-throughput study on the sensitivity of QTAIM to geometry and density level of theory, as well as provide the basis for geometric machine learning with GNNs. We refer to the collection of these three datasets with corresponding QTAIM information as the tmQM+ dataset Table 1.

**Table 1:** Three tiers of tmQM+ for QTAIM

Level of theory	Geometry optimization	Single point for QTAIM
‘Low’	xTB^a^	TPSS-D3BJ/def2-SVP
‘Mid’	PBE-D3BJ/def2-SVP^b^	TPSS-D3BJ/def2-SVP
‘High’	PBE-D3BJ/def2-SVP^b^	PBE0-D3BJ/def2-TZVP

a Geometries from tmQM.^31^

b Geometries from tmQMg.^32^

## Results and discussion

### Robustness of QTAIM to level of theory

**Fig. 1:**
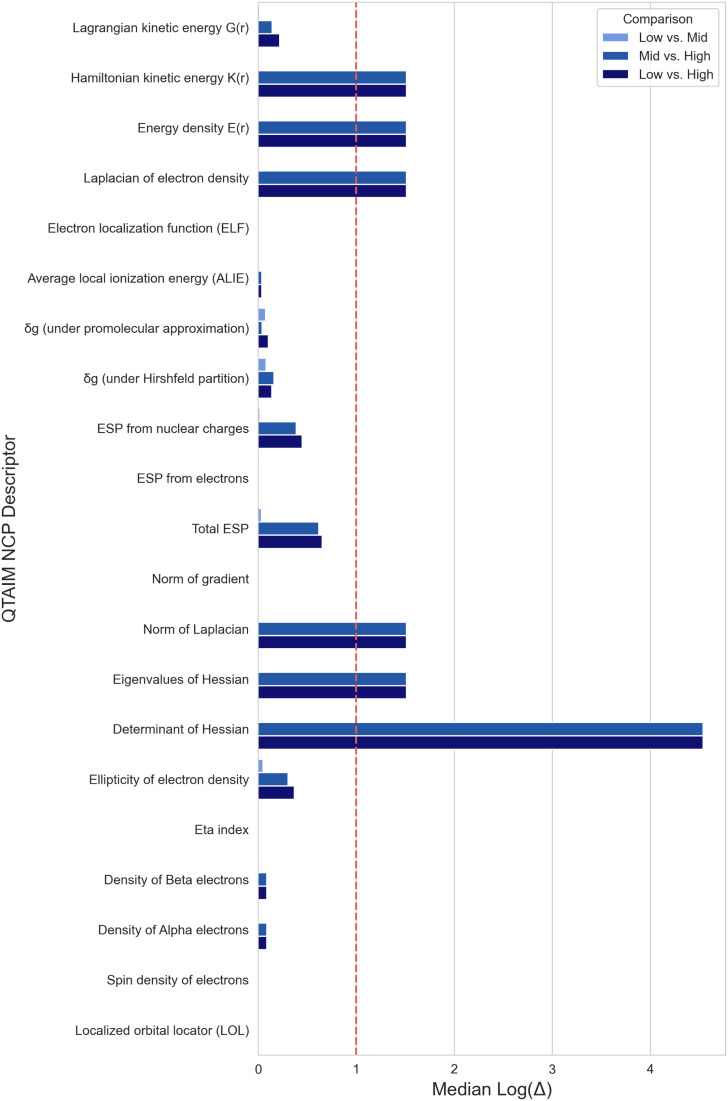
Median log difference of QTAIM Nuclear Critical Point descriptors between tmQM+ datasets.

The structure of the tmQM+ dataset affords the ability for large scale comparison of QTAIM descriptors and bonding interactions at different LOT. Our analysis shows that nuclear critical point (CP) descriptors are highly consistent at the same level of theory for density, regardless of the method of geometry optimization (Low *vs.* Mid, Fig. 1). Changing the density level of theory introduces variability in the descriptors whether the geometry differs or not (Mid *vs.* High, Low *vs.* High, Fig. 1). Certain nuclear CP descriptors are more sensitive to changes in functional and basis set than others. The determinant of the Hessian has an especially large error due to summing the log errors in the Hessian eigenvalues. Overall, with a few significant exceptions, most nuclear critical point QTAIM descriptors are largely consistent across different levels of theory for geometry optimization and density single point calculations.

**Fig. 2:**
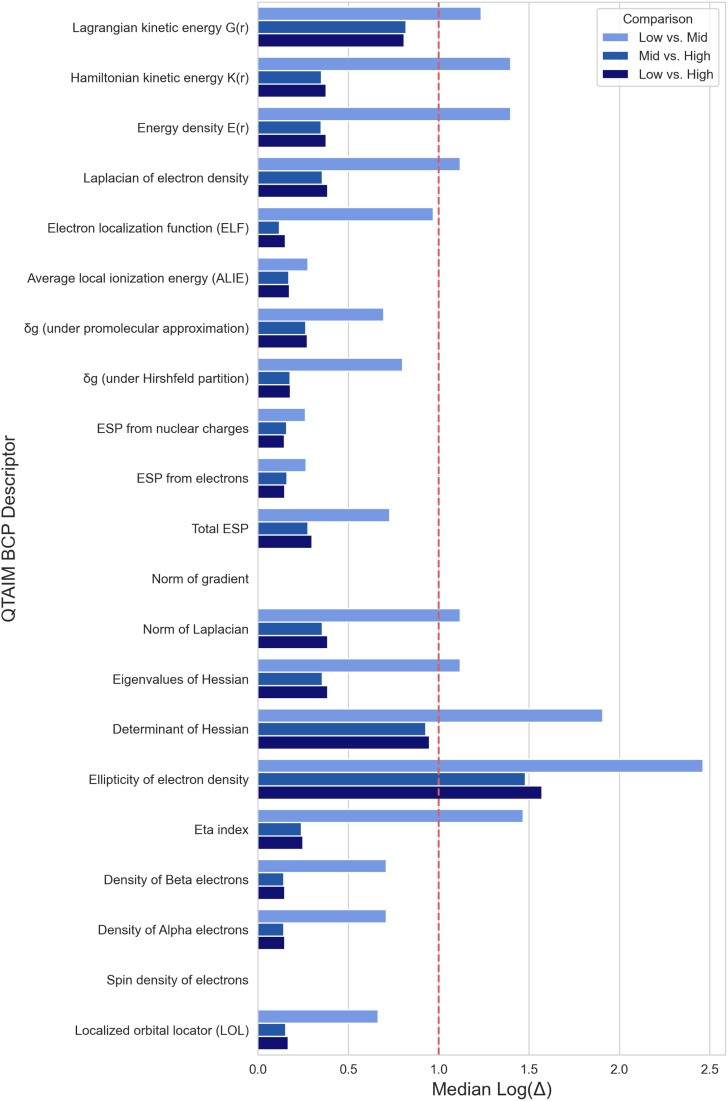
Median log difference of QTAIM Bond Critical Point descriptors between tmQM+ datasets.

Bond critical point features have greater variability for more features, but to a less extreme extent than the nuclear CP features (only ellipticity of electron density has a median log difference greater than 1) (Fig. 2). Bond CP features differ the most between geometries from different optimization methods (Low *vs.* Mid, Fig. 2). Changing the single point level of theory reveals less variability in the features (Mid *vs.* High, Low *vs.* High, Fig. 2).

**Fig. 3:**
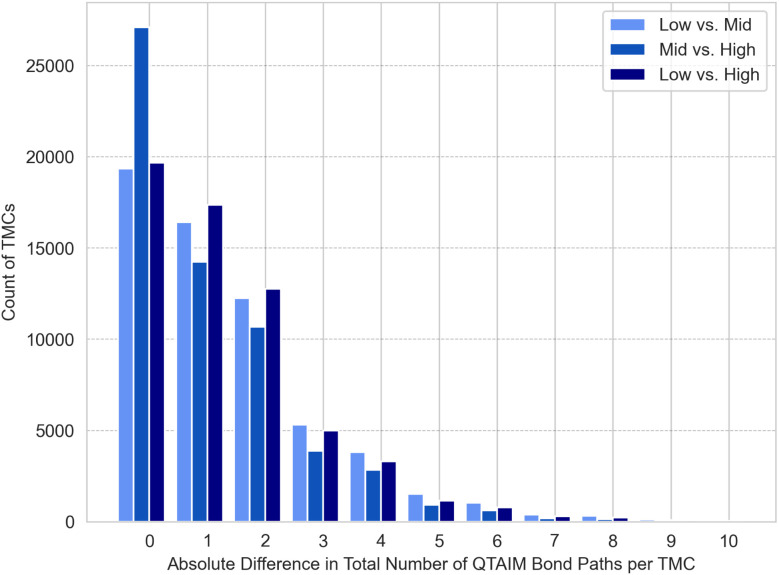
Count of transition metal complexes (TMCs) with absolute differences in total number of QTAIM bonding interactions per TMC between tmQM+ datasets.

For the bond interaction paths identified from densities at different levels of theory, we see that most often structures have the same number of bond paths regardless of the method for geometry optimization or single point (Fig. 3). More structures differ in the total number of bond paths when the geometry level of theory varies (Mid *vs.* High, Low *vs.* High, Fig. 3). The bonding interactions that tend to differ are non-covalent interactions. To illustrate with complexes that have a total number of bond paths differing by one between LOT, the complex C_12_H_16_As_2_HfS_3_ has two aromatic cyclopentadienyl ligands with variable bond paths identified from their carbons to the metal center (Fig. S1), while the complex C_32_H_40_Cl_2_P_4_Ru has an additional bond path between two hydrogens at the lower LOT (Fig. S2). Small differences in the density of weaker interactions can change whether the threshold for a bond path is met. Notably, the percentage of complexes that differ by at most 2 bonding interactions between levels of theory is 79.1% for ‘Low *vs.* Mid’, 85.7% for ‘Mid *vs.* High’, and 82.0% for ‘Low *vs.* High’, indicating that the core connectivity of most complexes is consistent.

### Overall machine learning performance

#### Formation energies

First, the performance between models at our lower and higher levels of QTAIM theory are marginally separated with less than 2% difference in % EwT (percentage of energies within threshold of chemical accuracy) and RMSEs. RMSE is particularly interesting as it is a statistic sensitive to outliers and has a diffference less than 0.5 meV per atom. Qualitatively, there are a few more extreme outliers in the low-level QTAIM model but we can conclude that for this prediction task, it is not worthwhile to generate more accurate geometries and densities. Interestingly, between the two models without QTAIM features, there are hardly any differences in performance. This suggests that more accurate geometries are not worth computing for formation energies on this dataset. Furthermore, the gap in performance between QTAIM and non-QTAIM models is small but clear across all metrics which may suggest that for larger datasets, QTAIM features are not necessarily going to yield improvement in performance and that higher levels of QTAIM theory do not necessarily yield improved ML models. Below we will mention use cases where QTAIM may improve model performance more dramatically Table 2.

**Table 2:** Model Performance on tmQMg Formation Energies (MAR, meV per Atom)

Model	*R* ^2^	MAE
Low – No QTAIM descriptors	0.967	0.209
Low – QTAIM descriptors	0.972	0.212
High – No QTAIM descriptors	0.964	0.227
High – QTAIM descriptors	0.982	0.155

#### Orbital energies

Observing our predicted HOMO, LUMO, and gap energies, we see the top model without QTAIM performed decently with only gap energies yielding MAEs above 10 mHa. Despite this, our models clearly benefited from the addition of QTAIM descriptors with gap energies, in particular, improving. The ‘Low’-LOT, QTAIM-informed model achieves sub-8 mHa gap energy prediction and the high LOT model achieves a notable sub-7 mHa MAE. Here the performance gap between QTAIM and non-QTAIM models is more substantial across all three label classes with, in particular, gap energies improving substantially between the classes of ML models with *vs.* without QTAIM features Table 3.

**Table 3:** Model Performance on tmQMg orbital energies (mHa)

Model	HOMO MAE	LUMO MAE	Gap MAE
Low – No QTAIM descriptors	7.6	8.6	10.5
Low – QTAIM descriptors	5.0	5.7	7.2
High – No QTAIM descriptors	8.2	8.6	10.4
High – QTAIM descriptors	4.9	6.1	6.8

#### Formation energy learning curves

Learning curves were constructed for the performance of models predicting formation energies trained upon datasets of size 50, 500, 5000, 10 000 and 50 000 transition metal complexes (Fig. S3). Models were built upon QTAIM connectivity with and without the set of QTAIM descriptors at both the ‘Low’ and ‘High’ LOT. For the smallest training sets (size 50 and 500), models exhibit very large RMSE, except for the model with ‘High’-level QTAIM descriptors which dramatically stabilize outlier predictions. Additionally, only a moderate percentage of complexes are predicted within chemical accuracy in this small-data regime. For larger training sets (size 5000 and 10 000), the addition of QTAIM descriptors affords consistent improvement in test metrics as they converge to the values of the full training set (size 50 000). The models with ‘Low’-level QTAIM descriptors achieve comparable RMSE and higher % EwT for training sets of size 10 000 and larger. Overall, QTAIM descriptors at both LOT improve model performance across the board on datasets from 1000 to 10 000 training points – the regime where GNNs generally become applicable.

### tmQM generalizability

Following our previous work where we showed that GNNs augmented with QTAIM descriptors showed superior generalizability across unseen charge domains,^19^ we sought to study this effect on the tmQM+ datasets. Here we use the two levels of theory to test whether cheaper QTAIM calculations could offer the same generalizability improvements for machine learning. Our domains for generalizability are charge and identity of the metal.

#### Charge

Our first test of generalizability centers on charge. Here we filter our training set to include only neutral species while filtering the test set to only include charged species with charge ∈{−1, 1}. This stratification mirrors our previous study^19^ and we report models trained on formation energies and separately on orbital energies. Filtering these datasets yields a training set of 40 000 and test set of 2000 data points, respectively.

**Fig. 4:**
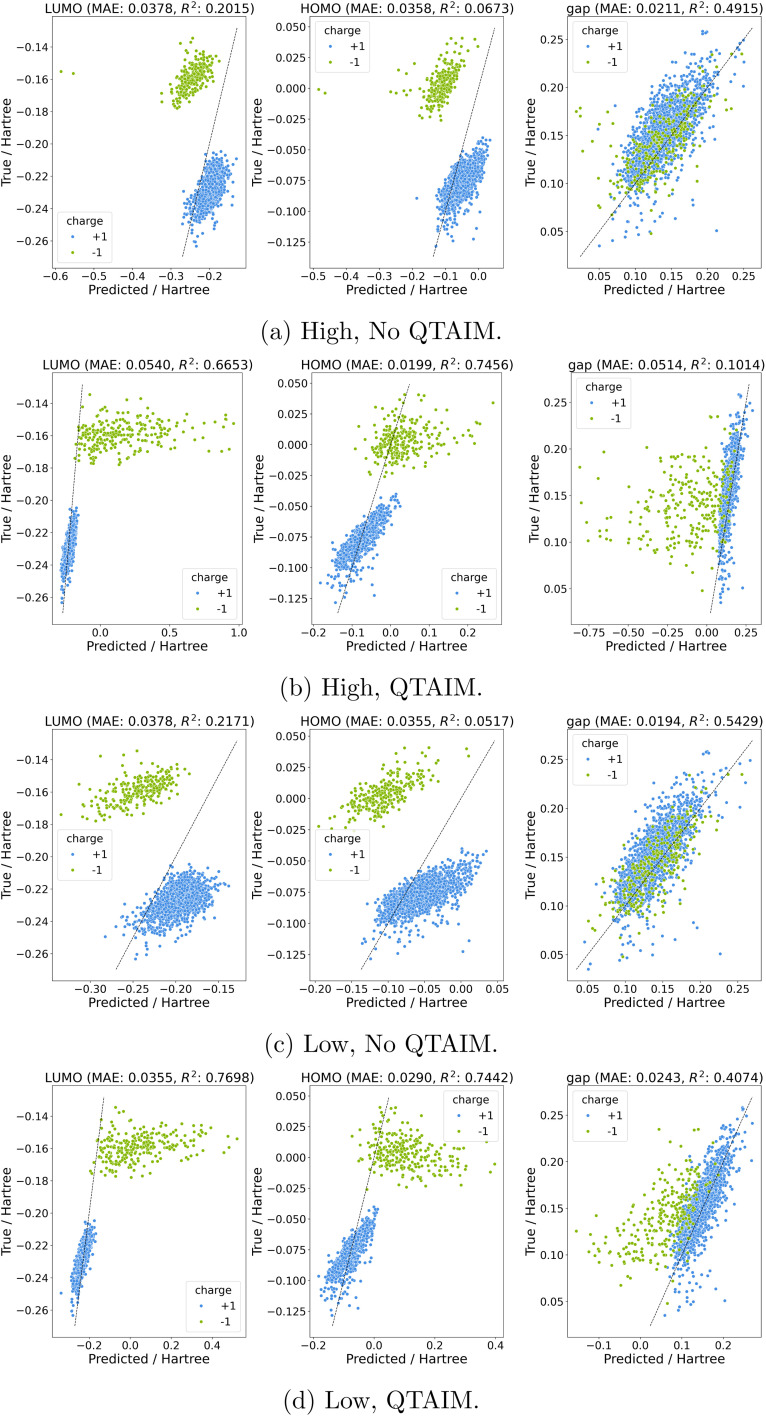
Charge Out-of-domain prediction of orbital energies for (a) high without QTAIM descriptors, (b) high with QTAIM descriptors, (c) low without QTAIM descriptors, and (d) low with QTAIM descriptors. Note that nine structures were removed as a result of being extreme outlier predictions in either (a) or (c); see SI for details.

Between the higher and lower level of theory datasets we see a similar narrative emerge where the inclusion of QTAIM features leads to more stable predictions with less extreme outliers in predictions. Models trained on formation energies lead to mixed results with QTAIM models containing fewer strong outliers but slightly worse overall performance (Fig. S10). Orbital energies, on the other hand, show a clear victory for QTAIM features (Fig. 4). Here predicted HOMO and LUMO energies in the test set show a systematic shift *versus* true values. This is hardly surprising given the distribution of orbital energies at each charge where HOMO and LUMO distributions have markedly different means at different charge states (Fig. S4 and S5).

#### Metal identity

**Fig. 5:**
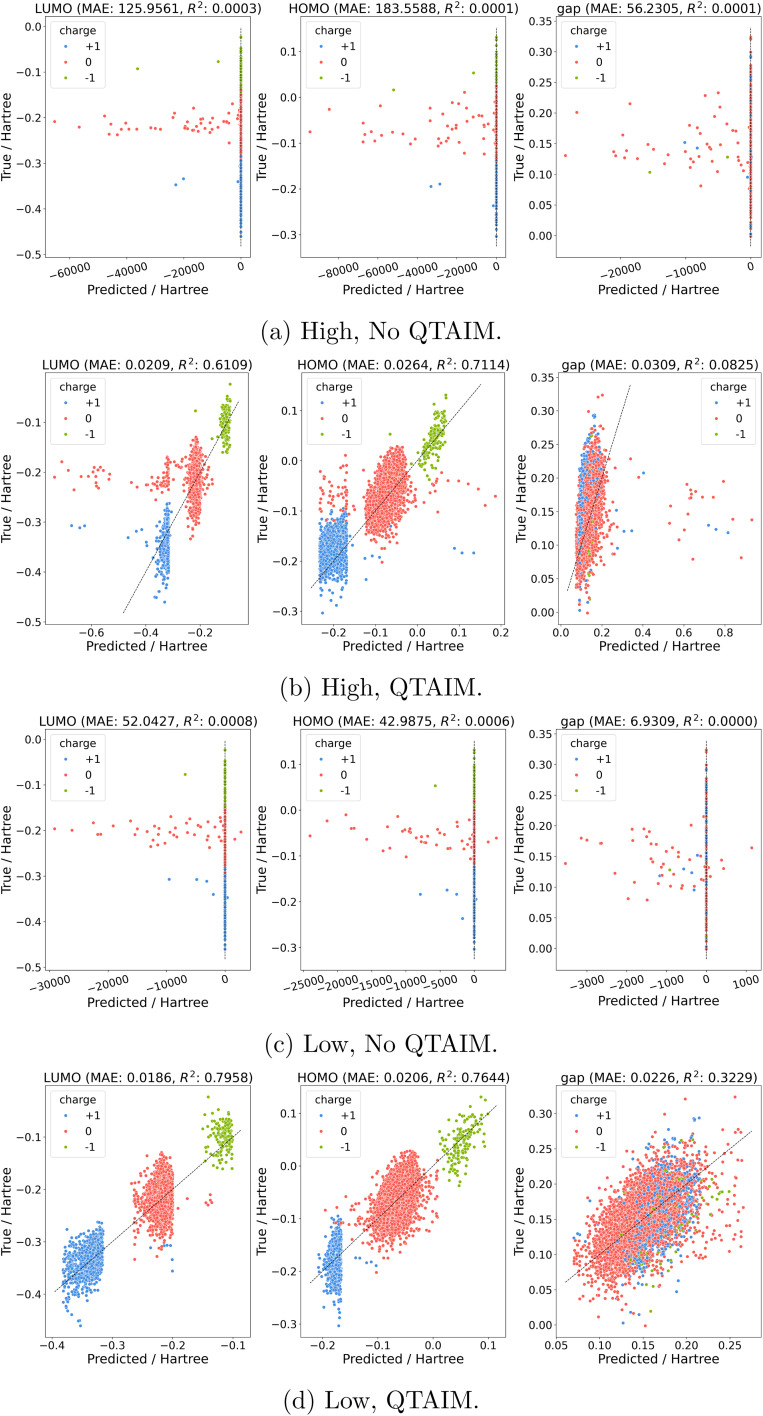
Metal out-of-domain prediction of orbital energies for (a) high without QTAIM descriptors, (b) high with QTAIM descriptors, (c) low without QTAIM descriptors, and (d) low with QTAIM descriptors. Note that six structures were removed as a result of being extreme outlier predictions in either (a) or (c); see SI for details.

The tmQM dataset includes metals across the entire d block, offering a diverse set of elements that's quite rare for published molecular property datasets. This allowed us to include certain elements in training and test sets and study whether QTAIM could improve predictions of unseen elements. Heuristically, we expect quantum features from QTAIM to act as an alternative, rich featurization scheme for atoms compared to traditional methods such as one-hot encoding.^48,49^ We filtered the training set to remove all molecules with any element beyond Kr, limiting the training set to first row transition metal complexes. The test set was filtered to remove complexes where every atom was below this threshold. This partitioning yields sets of around 17 000 training points and just under 8000 testing points. With this train-test set, we only predicted orbital energies as these frontier orbitals are generally more sensitive to the transition metals in these complexes, whereas formation energies are a function of every atom in the molecules. Models with lower-level geometries and QTAIM calculations show similar results to the charge out-of-domain testing with QTAIM stabilizing outliers while trading off some performance for in-domain data (Fig. 5). On the other hand, high-level QTAIM/geometries show a massive performance gap between QTAIM and non-QTAIM models. Here QTAIM models are able to significantly extrapolate to OOD elements while non-QTAIM models perform quite poorly. The later fact suggests that higher-level QTAIM connectivity, alone, cannot offer these extrapolation benefits, but that inclusion of QTAIM features can help train models that are more robust to extrapolation out-of-domain.

## Conclusion

Herein we predict properties of transition metal complexes of different charges with graph neural networks built upon QTAIM connectivity and descriptors. We perform extensive computational studies on our three-tier tmQM+ dataset, including standard model training/testing, out-of-domain studies, and learning curves, as well as a comparison of QTAIM descriptors across different levels of theory (LOT) for geometries and electron densities. This rich analysis is notable as we aim to address: (1) how stable is QTAIM across different LOT? and (2) how do quantum features, in this case QTAIM, empower machine learning across different LOT?

This first portion represents the first, to our knowledge, high-throughput benchmark of QTAIM features for transition metal complexes at different LOTs. We find that density LOT has a large effect on some nuclear critical point QTAIM values, while the geometry LOT has a negligible effect. Conversely, geometry LOT is more influential for bond critical point QTAIM values. We also find that QTAIM bonding interactions are largely stable across both geometry and density LOT with 80% of complexes maintaining the same number of bond paths ±2.

Towards the latter question, we conducted model testing on both formation and orbital energies. With standard training/testing on the complete datasets, we found that QTAIM descriptors offered marginal improvements at both levels of theory for formation energies, but notable improvements for predicting orbital energies. Learning curves showed that QTAIM descriptors offer meaningful model improvements, especially towards outliers, across datasets of at least 5000 training points. Finally, by stratifying across charge and metal identity, we determined that QTAIM can substantially improve out-of-domain predictions, especially at higher LOT.

This study contributes to guiding scientists seeking to incorporate quantum mechanical descriptors in machine learning models. We find that, in most cases, descriptors obtained at a lower level of theory offer similar benefits to more expensive quantum calculations for machine learning. In particular, this approach is beneficial for stabilizing outliers and enabling existing models to make predictions in unseen chemical domains. This approach also improves training performance for smaller datasets. We hope that the field will perform more exhaustive benchmarking and build on these findings. Important questions remain such as whether ML-predicted quantum features can offer similar benefits for outlier and out-of-domain predictions. This is especially relevant as more datasets and models are released for predicting quantum chemical features and using them for downstream tasks.^50,51^ In addition, these quantum descriptor algorithms need evaluation for their universality and transferability across chemical domains and levels of theory.

## Conflicts of interest

The authors declare no conflict of interest.

## Supplementary Material

DD-004-D5DD00220F-s001

## Data Availability

Data for this article including train/test splits with labels (formation energies and orbital energies) and QTAIM descriptors are available at https://doi.org/10.6084/m9.figshare.29099921.v1. The code for both generating QTAIM values and training models can be found with https://figshare.com/articles/software/Static_code_repository_for_Multi-level_Quantum-Informed_Graph_Neural_Networks_for_Resolving_Properties_of_Transition_Metal_Complexes_/30246229 at 10.6084/m9.figshare.30246229. Supplementary information: notable QTAIM structures, a table of QTAIM descriptors used, label statistics, and outlier. See DOI: https://doi.org/10.1039/d5dd00220f.
