# Supplementary material for: Multi-level QTAIM-enriched graph neural networks for resolving properties of transition metal complexes
Source: Digit Discov. 2025 Oct 15;4(11):3378–88. doi: 10.1039/d5dd00220f (PMC12538384; doi:10.1039/d5dd00220f)
Supplement: DD-004-D5DD00220F-s001 [file DD-004-D5DD00220F-s001.pdf]

Supplementary Information (SI) for Digital Discovery.
This journal is © The Royal Society of Chemistry 2025

Supporting Information: Multi-level QTAIM-Enriched Graph Neural Networks for Resolving Properties of Transition Metal Complexes

Winston Gee,[†,‖] Santiago Vargas,[*,†,‡,‖] Abigail Doyle,[†] and Anastassia N. Alexandrova[*,†,¶,§]

†*Department of Chemistry and Biochemistry, University of California, Los Angeles, Los Angeles, California 90095 USA.*

‡*Current address: Chemical Sciences Division, Lawrence Berkeley National Laboratory, 1 Cyclotron Rd, Berkeley, CA 94720*

¶*Department of Materials Science and Engineering, University of California, Los Angeles, California 90095 USA.*

§*California NanoSystems Institute, Los Angeles, California 90095 USA.*

‖*These Authors Contributed Equally*

E-mail: santiagovargas921@gmail.com; ana@chem.ucla.edu

Contents

List of Tables

S1 QTAIM Descriptors Obtained at Nuclear Critical Points (NCP) and Bond Critical Points (BCP). S-4

S2 Removed Outliers of Charge Out-of-Domain Prediction of Orbital Energies . S-8

S3 Removed Outliers of Metal Out-of-Domain Prediction of Orbital Energies . . S-8

List of Figures

S1 Connectivity from QTAIM for $C_{12}H_{16}As_2HfS_3$ at a) high and b) low level of theory (edges indicated by silver rods between atoms when a QTAIM bonding interaction is present). Note the variability in bonding interactions between the carbons of the aromatic cyclopentadienyl ligands and Hf (light blue). . . S-5

S2 Connectivity from QTAIM for $C_{32}H_{40}Cl_2P_4Ru$ at a) high and b) low level of theory (edges indicated by silver rods between atoms when a QTAIM bonding interaction is present). Note the single additional H-H interaction present (at center, H in pink) at the lower level of theory. S-5

S3 Learning curves for Formation Energies with a) Test RMSE and b) Test $\%EwT$ (Energy within Threshold of Chemical Accuracy) for training datasets of size 50, 500, 5000, 10000, and 50000 transition metal complexes. S-6

S4 Distribution of Formation Energies by Charge of Transition Metal Complex . S-7

S5 Distribution of Orbital Energies by Charge of Transition Metal Complex . . S-7

S6 Charge Out-of-Domain Prediction of Orbital Energies including outliers listed in S2 . . . . S-9

S7 Removed Outliers of Charge Out-of-Domain Prediction of Orbital Energies . S-10

S8 Metal Out-of-Domain Prediction of Orbital Energies including outliers listed in S3. . . . . S-11
S9 Removed Outliers of Metal Out-of-Domain Prediction of Orbital Energies . S-12
S10 Charge Out-of-Domain Prediction of Formation Energies for S11a) High No QTAIM Descriptors, S11b) High QTAIM Descriptors, S11c) Low No QTAIM Descriptors, and S10d) Low QTAIM Descriptors. S-13
S11 Metal Out-of-Domain Prediction of Formation Energies for S8a) High No QTAIM Descriptors, S8b) High QTAIM Descriptors, S8c) Low No QTAIM Descriptors, and S11d) Low QTAIM Descriptors. S-14

Erroneous Structures

We have identified additional erroneous structures similar to those identified by Garrison et al. These structures involve carbons that lack hydrogen due to the construction of the tmQM dataset from crystal structures in the Cambridge Structural Database. The additionally identified structures are provided in an ase database.

Descriptors from QTAIM Analysis

Table S1: QTAIM Descriptors Obtained at Nuclear Critical Points (NCP) and Bond Critical Points (BCP).

**QTAIM Descriptors**	
Lagrangian kinetic energy G(r)	Norm of gradient
Hamiltonian kinetic energy K(r)	Norm of Laplacian
Energy density E(r)	Eigenvalues of Hessian
Laplacian of electron density	Determinant of Hessian
Electron localization function (ELF)	Ellipticity of electron density
Average local ionization energy (ALIE)	Eta index
δg (under promolecular approximation)	Density of Beta electrons
δg (under Hirshfeld partition)	Density of Alpha electrons
ESP from nuclear charges	Spin density of electrons
ESP from electrons	Localized orbital locator (LOL)
Total ESP	

Graph Connectivity from QTAIM Analysis

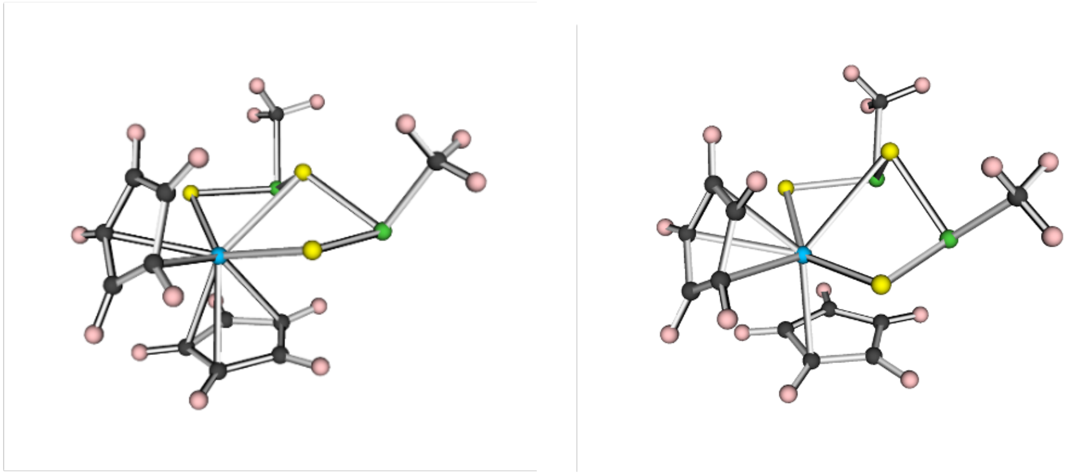

(a) (b)

Figure S1: Connectivity from QTAIM for $C_{12}H_{16}As_2HfS_3$ at a) high and b) low level of theory (edges indicated by silver rods between atoms when a QTAIM bonding interaction is present). Note the variability in bonding interactions between the carbons of the aromatic cyclopentadienyl ligands and Hf (light blue).

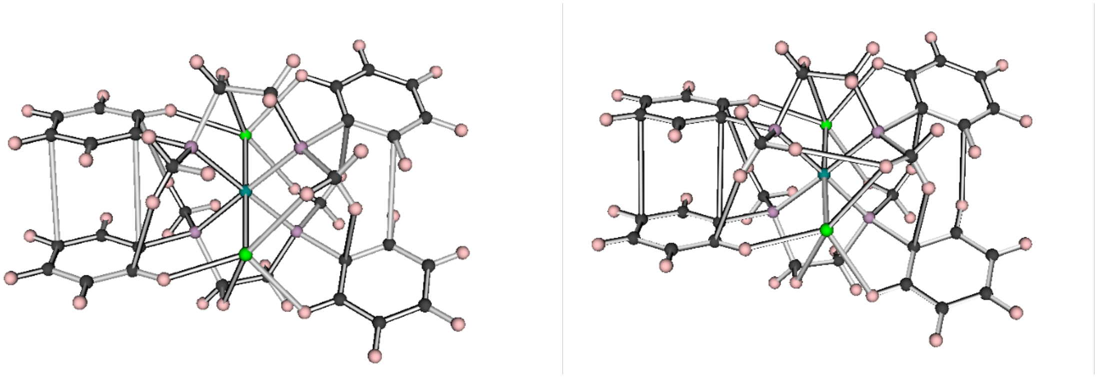

(a) (b)

Figure S2: Connectivity from QTAIM for $C_{32}H_{40}Cl_2P_4Ru$ at a) high and b) low level of theory (edges indicated by silver rods between atoms when a QTAIM bonding interaction is present). Note the single additional H-H interaction present (at center, H in pink) at the lower level of theory.

Learning Curves

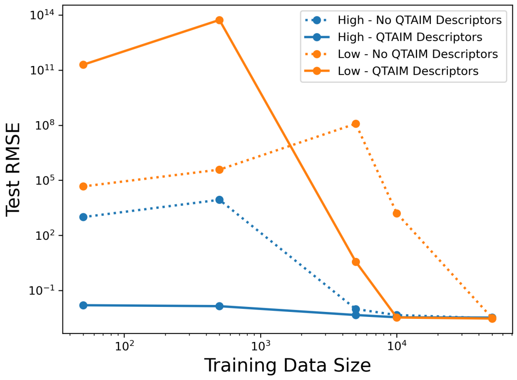

(a)

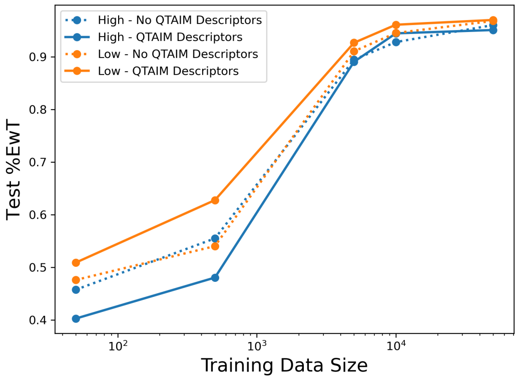

(b)

Figure S3: Learning curves for Formation Energies with a) Test RMSE and b) Test $\%EwT$ (Energy within Threshold of Chemical Accuracy) for training datasets of size 50, 500, 5000, 10000, and 50000 transition metal complexes.

Distribution of Energies by Charge

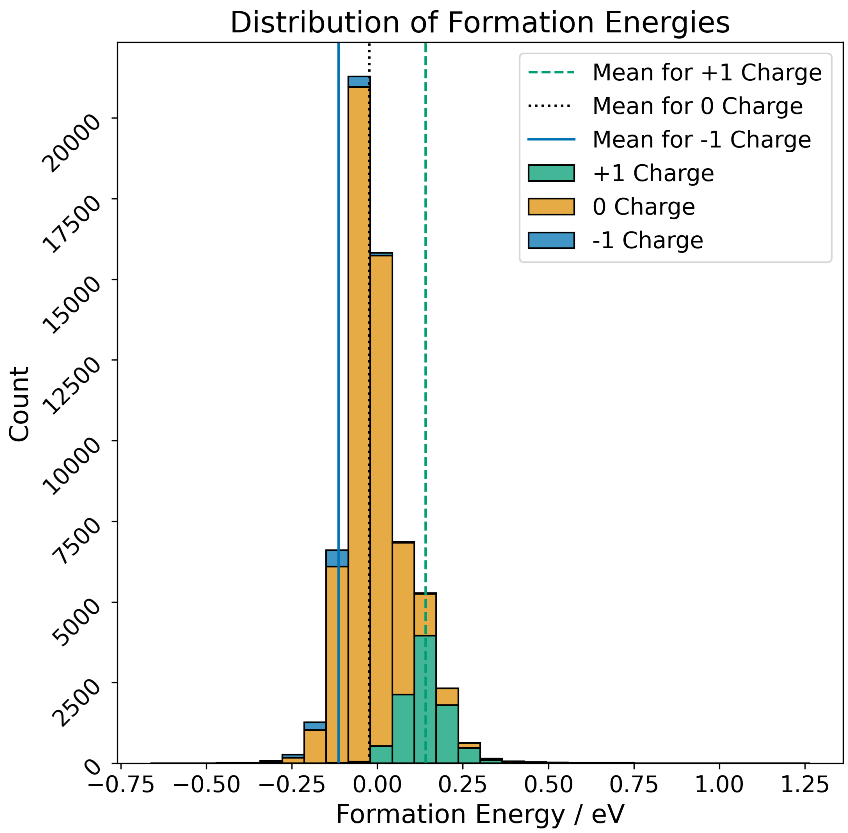

Figure S4: Distribution of Formation Energies by Charge of Transition Metal Complex

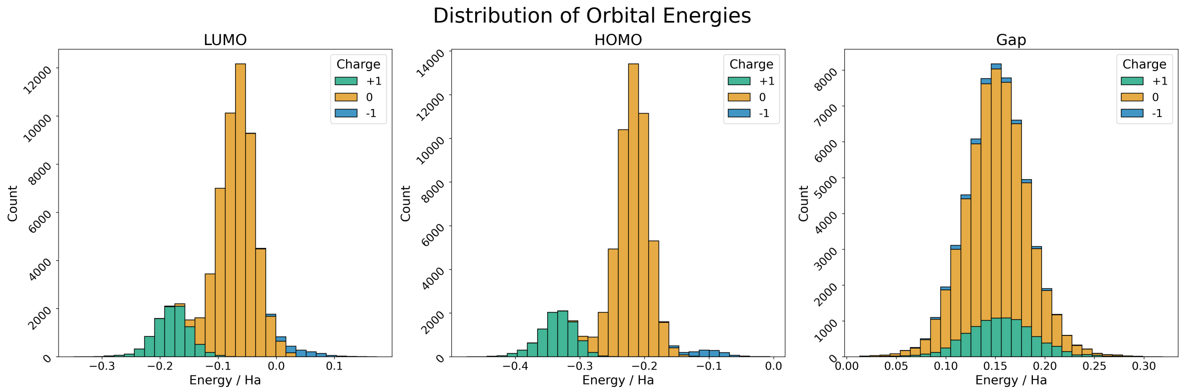

Figure S5: Distribution of Orbital Energies by Charge of Transition Metal Complex

Outliers Removed from Out-of-Domain Prediction of Orbital Energies

Table S2: Removed Outliers of Charge Out-of-Domain Prediction of Orbital Energies

ids	charge	lumo preds	homo preds	gap preds	lumo labels	homo labels	gap labels
RULMOA	-1	-55761.835	-64573.031	-34723.277	-0.178320	-0.0204545	0.1414350
PUCWEQ	-1	-20508.166	-23665.632	-12902.361	-0.177008	-0.0075088	0.1685067
BEKMIP	-1	-9576.3310	-9643.0253	-4196.7060	-0.1680334	0.0002719	0.1597456
AQEPIV	-1	-23.891029	5.5616583	24.386741	-0.1552922	0.0242006	0.1782422
NENOXC	-1	-7051.7431	-4372.2631	1350.3457	-0.1635314	0.0120665	0.1743001
JONNUV	-1	359.94201	-1058.9995	-1507.659	-0.1729178	0.0014807	0.1775858
GIKJOB	-1	-3.4527401	-4.0114970	-0.3226882	-0.1725499	-0.0078125	0.1541464
QIBFEP	-1	-3.336441	-4.056635	-0.5629981	-0.1576244	0.0133343	0.1592843
MENWUA	-1	1407.6134	-3866.0661	-5689.4482	-0.1720835	0.0228452	0.2263338

Table S3: Removed Outliers of Metal Out-of-Domain Prediction of Orbital Energies

ids	charge	lumo preds	homo preds	gap preds	lumo labels	homo labels	gap labels
KIGKUG	0	-140667.82	-202860.79	-60860.742	-0.2105388	-0.0847338	0.1219798
EDIGUT	0	-263919.34	-377443.65	-110956.03	-0.226383	-0.0440923	0.1870156
RATDUM	0	-412280.25	-584148.6	-166891.76	-0.2448796	-0.0894075	0.155923
BIZQIK	0	-164573.04	-237204.89	-71076.695	-0.2153468	-0.0873058	0.124519
CPAINC	0	-58329.703	-21242.148	25249.662	-0.2202555	-0.1013667	0.1139210
HESGOE	0	16998.429	12198.234	-18134.318	-0.2275429	-0.1043090	0.11887408

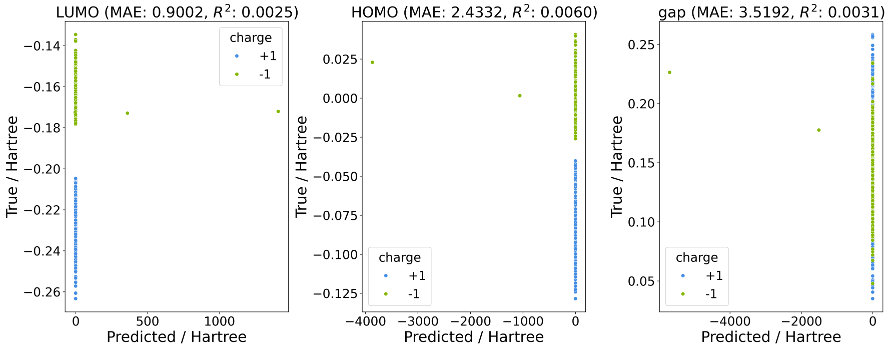

(a) High, No QTAIM.

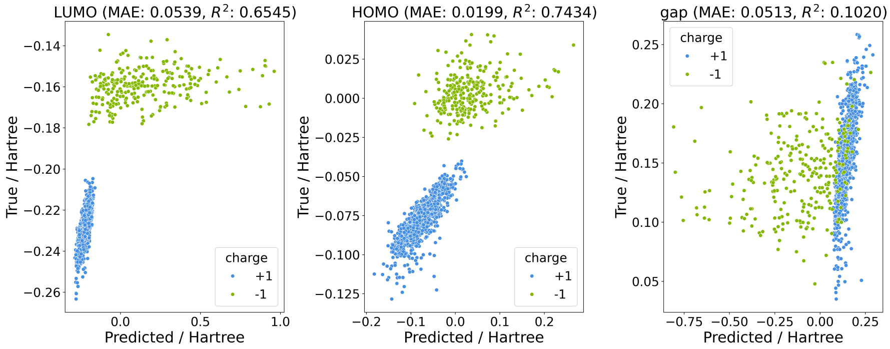

(b) High, QTAIM.

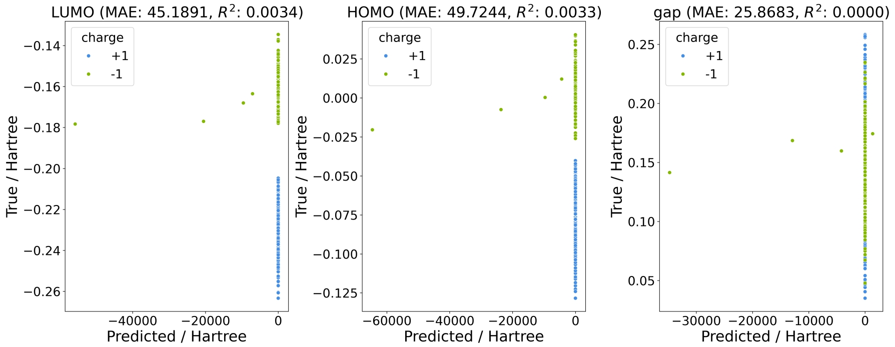

(c) Low, No QTAIM.

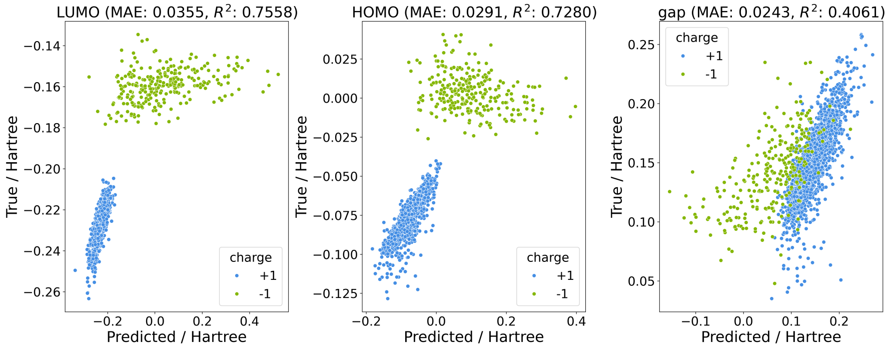

(d) Low, QTAIM.

Figure S6: Charge Out-of-Domain Prediction of Orbital Energies including outliers listed in S2

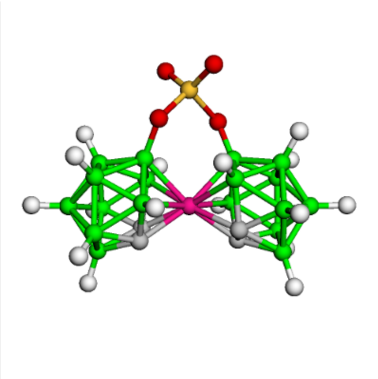

(a) RULMOA ($C_4H_{16}B_{18}CoO_4S$)

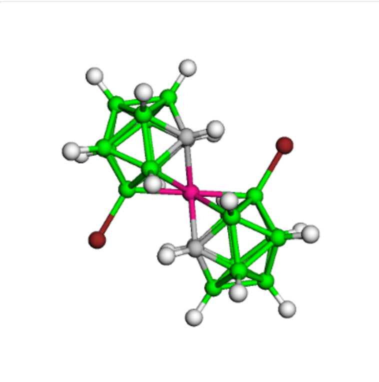

(b) PUCWEQ ($C_4H_{20}B_{18}Br_2Co$)

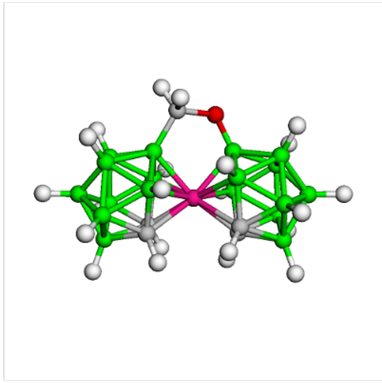

(c) BEKMIP ($C_5H_{22}B_{18}CoO$)

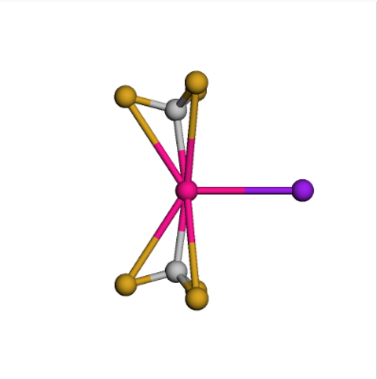

(d) AQEPIV (C_2F_6HgI)

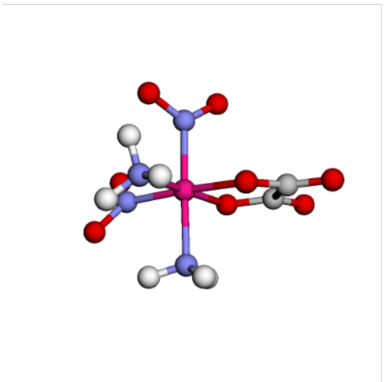

(e) NENOXC ($C_2H_6CoN_4O_8$)

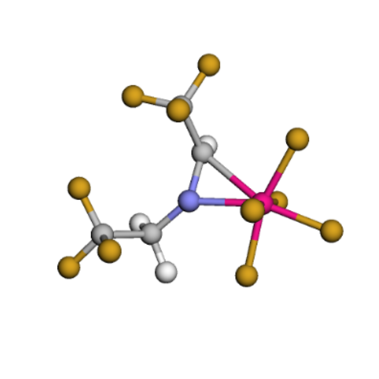

(f) JONNUV ($C_4H_3F_{11}NW$)

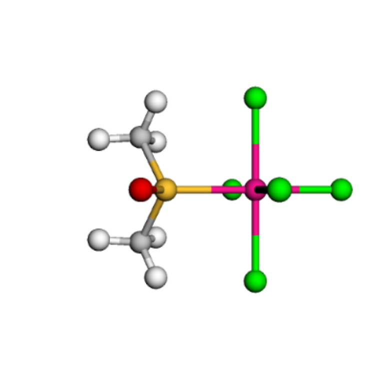

(g) GIKJOB ($C_2H_6Cl_5OPtS$)

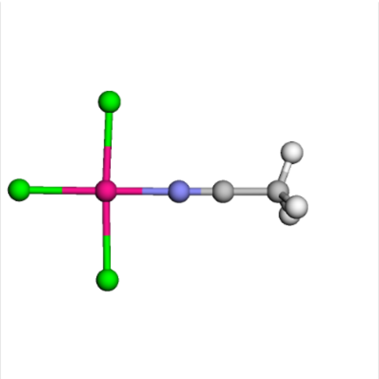

(h) QIBFEP ($C_2H_3Cl_3NPd$)

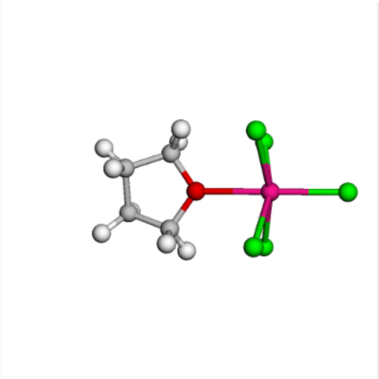

(i) MENWUA ($C_4H_8Cl_5HfO$)

Figure S7: Removed Outliers of Charge Out-of-Domain Prediction of Orbital Energies

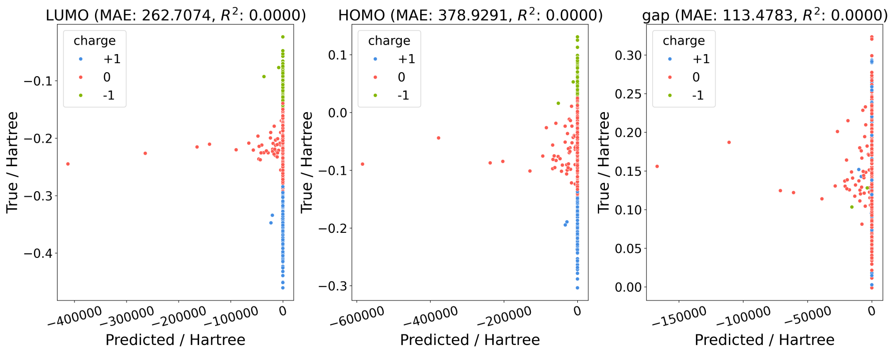

(a) High, No QTAIM.

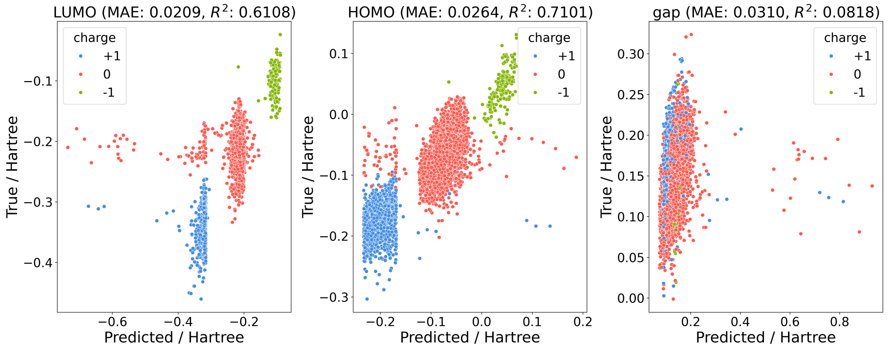

(b) High, QTAIM.

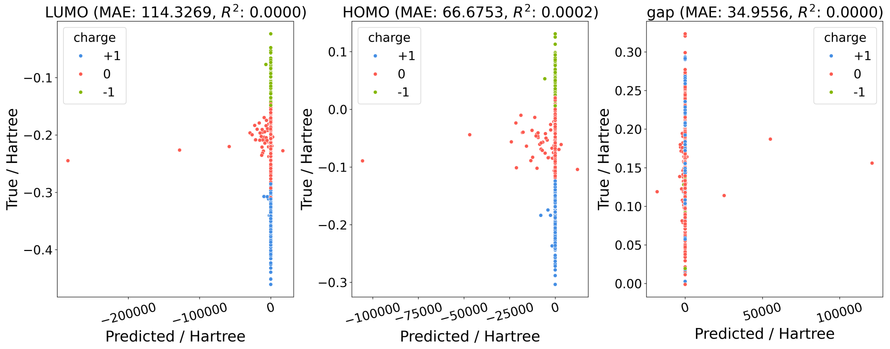

(c) Low, No QTAIM.

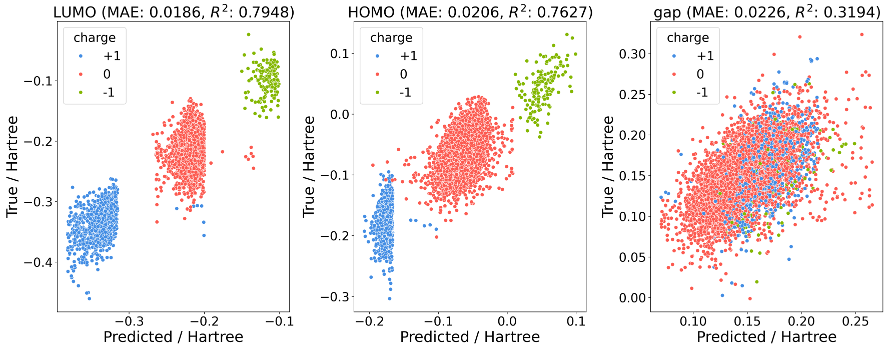

(d) Low, QTAIM.

Figure S8: Metal Out-of-Domain Prediction of Orbital Energies including outliers listed in S3.

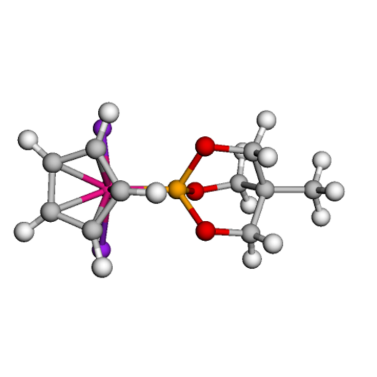

(a) KIGKUG ($C_{10}H_{14}CoI_2O_3P$)

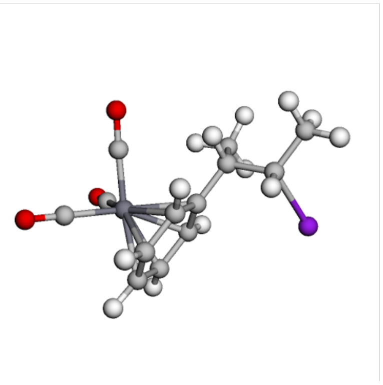

(b) EDIGUT ($C_{13}H_{13}CrIO_3$)

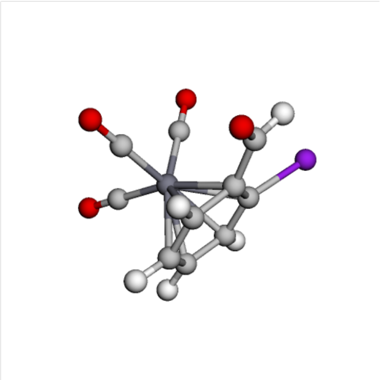

(c) RATDUM ($C_{10}H_5CrIO_4$)

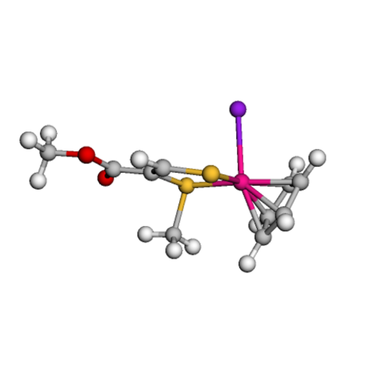

(d) BIZQIK ($C_{10}H_{12}CoIO_2S_2$)

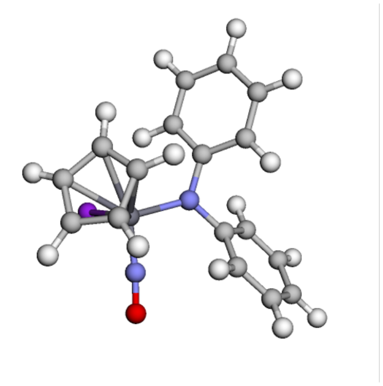

(e) CPAINC ($C_{17}H_{15}CrIN_2O$)

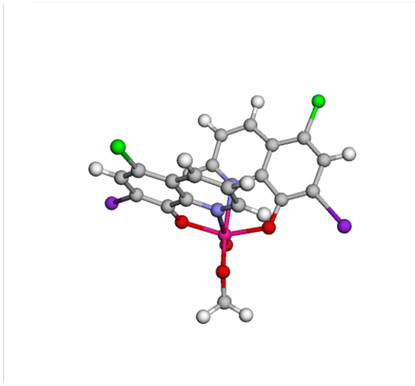

(f) HESGOE ($C_{19}H_{11}Cl_2I_2N_2O_4V$)

Figure S9: Removed Outliers of Metal Out-of-Domain Prediction of Orbital Energies

Out-of-Domain Prediction of Formation Energies

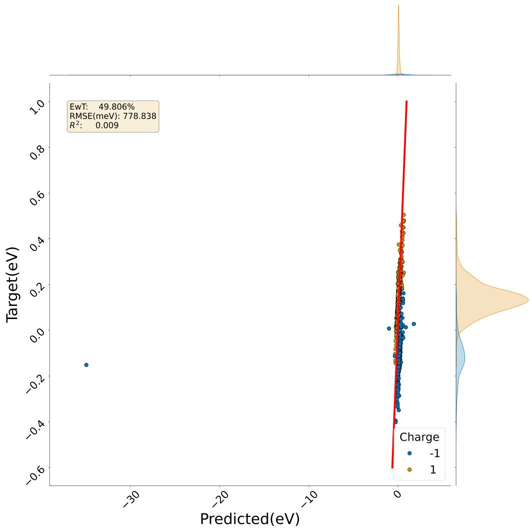

(a) High No QTAIM.

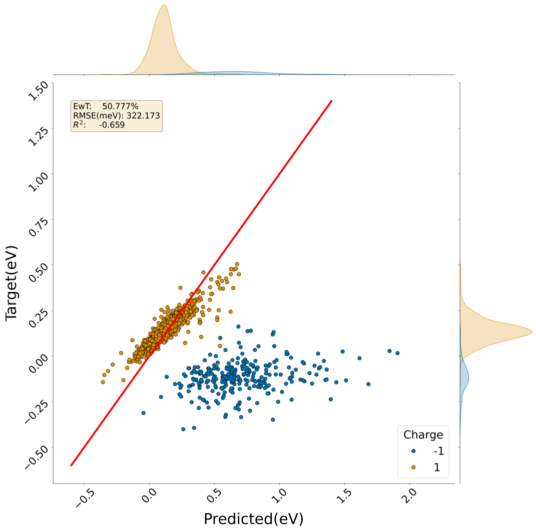

(b) High QTAIM.

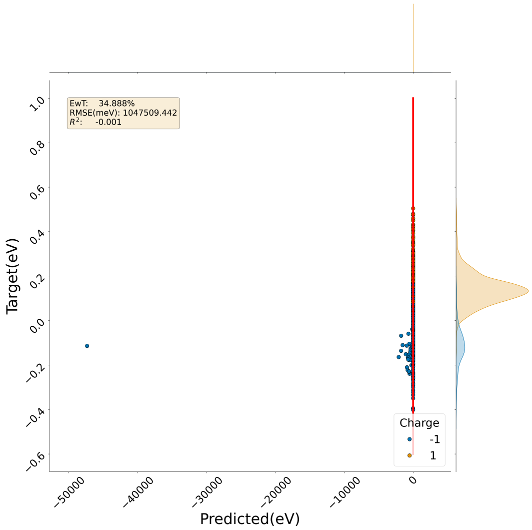

(c) Low No QTAIM.

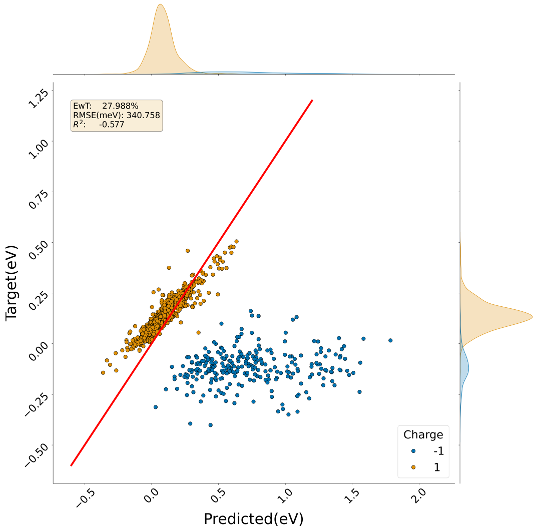

(d) Low QTAIM.

Figure S10: Charge Out-of-Domain Prediction of Formation Energies for S11a) High No QTAIM Descriptors, S11b) High QTAIM Descriptors, S11c) Low No QTAIM Descriptors, and S10d) Low QTAIM Descriptors.

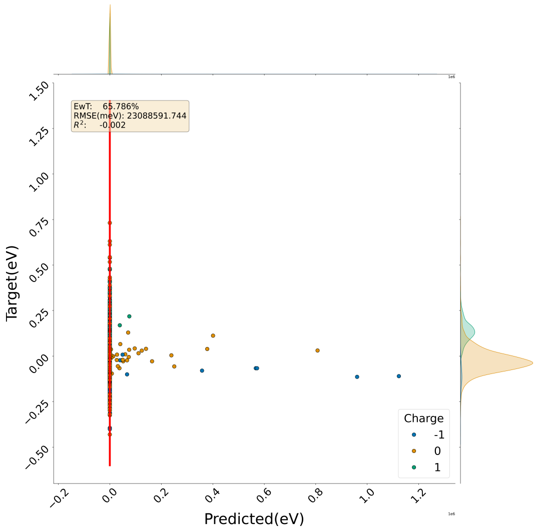

(a) High No QTAIM.

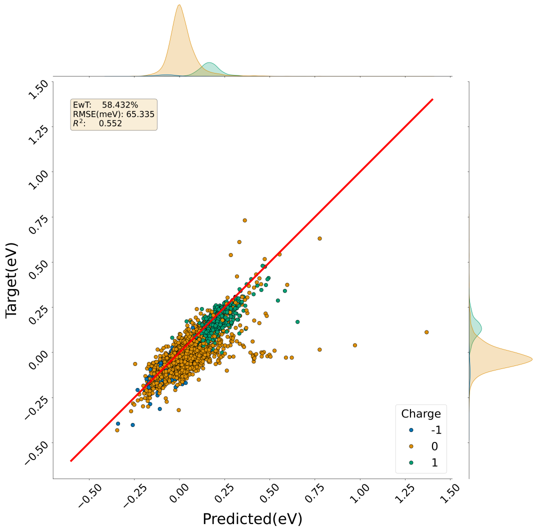

(b) High QTAIM.

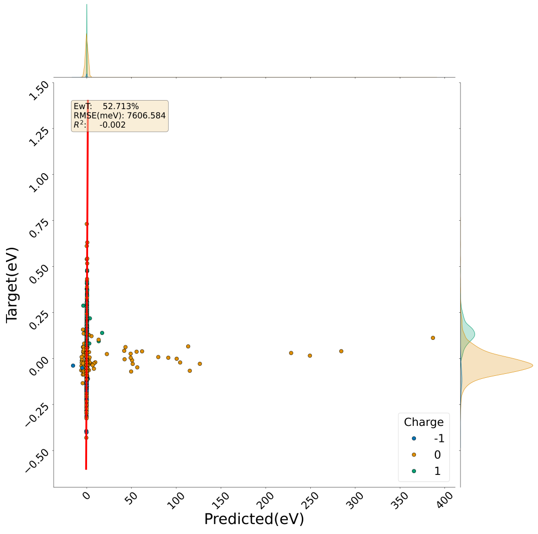

(c) Low No QTAIM.

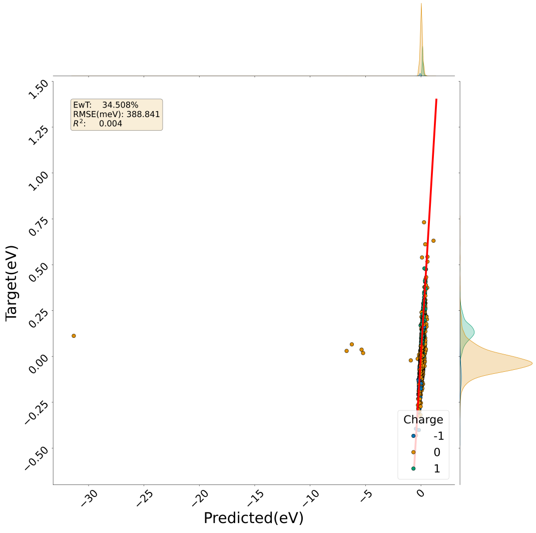

(d) Low QTAIM.

Figure S11: Metal Out-of-Domain Prediction of Formation Energies for S8a) High No QTAIM Descriptors, S8b) High QTAIM Descriptors, S8c) Low No QTAIM Descriptors, and S11d) Low QTAIM Descriptors.
